# Depression in the Perinatal Period: Course and Outcome of Depression in the Period from the Last Trimester of Pregnancy to One Year after Delivery in Primiparous Mothers

**DOI:** 10.3390/medicina60060970

**Published:** 2024-06-12

**Authors:** Olivera Zikic, Jelena Stojanov, Jelena Kostic, Gordana Nikolic, Suzana Tosic Golubovic, Maja Simonovic, Vladimir Djordjevic, Iva Binic

**Affiliations:** 1 Faculty of Medicine, Department of Psychiatry with Medical Psychology, University of Niš, Bul. dr Zorana Djindjića 81, 18000 Niš, Serbia; jelena.kostic@medfak.ni.ac.rs (J.K.); gordana.nikolic@medfak.ni.ac.rs (G.N.); suzana.tosic.golubovic@medfak.ni.ac.rs (S.T.G.); maja.simonovic@medfak.ni.ac.rs (M.S.); vladimir.djordjevic@medfak.ni.ac.rs (V.D.); ivabinic@gmail.com (I.B.); 2 Center for Mental Health Protection, University Clinical Center Nis, Bul. dr Zorana Djindjića 48a, 18000 Niš, Serbia; 3 Special Hospital for Psychiatric Diseases “Gornja Toponica”, 18202 Niš, Serbia; jelena.a.86.ja@gmail.com; 4 Clinic for Psychiatry, University Clinical Center Nis, Bul. dr Zorana Djindjića 48a, 18000 Niš, Serbia

**Keywords:** perinatal period, depression, prevalence, course, primiparous woman

## Abstract

*Background and Objectives*: One of the most significant psychiatric problems in women is depression related to the perinatal period. Our study aims to determine the frequency and course of depressive symptomatology in the perinatal period with particular reference to objective rate and outcome of postpartum depression. *Materials and Methods*: One hundred and eighty-eight pregnant/postnatal women were included in a prospective, longitudinal, observational study during which the depressive symptomatology was estimated at the third trimester of pregnancy, and the first, sixth, and twelfth month‚ postpartum. All participants completed a semi-structured sociodemographic questionnaire constructed for research purposes, the Edinburgh Postnatal Depression Scale, Toronto Alexithymia Scale, Beck Anxiety Inventory, and The Mood Disorder Questionnaire at each time point. Postpartum depression diagnosis was confirmed by a trained and certified psychiatrist with long-standing experience. For a better understanding of the trajectory of depressive symptomatology and genuine postpartum depression, we classified depression into those with new-onset and those left over from the previous observation period. *Results:* In general, 48.9% of participants in the study were depressed at some point during the investigation. A total of 10.6% of women were depressed in the third trimester. The highest percentage of new-onset depression (25%) was in the first month after giving birth and was maintained for up to six months, after which the appearance was sporadic. Most of the postpartum depression resolved in the period from the first month to the sixth month after childbirth (20.7%). The episodes mainly had characteristics of unipolar depression. *Conclusions:* Our results imply that a new onset of depression is most intensive during the first six months, and after that, it is sporadic. Further studies are needed to explore whether all depressive symptomatology in the postnatal period is the same, or perhaps postpartum depression, classified in this way, has specific characteristics, etiology, and consequently different treatment and preventive options.

## 1. Introduction

Depression in the perinatal period is a common mood disorder in women worldwide [1]. Most of the available literature defines perinatal depression as a mild or major non-psychotic depressive episode detected during pregnancy.

According to the systematic review of systematic reviews, the overall prevalence of perinatal depression is up to 26.3%, slightly higher during pregnancy, 28.5%, and postnatally, 27.6% [1]. Depending on the detection method, a higher rate was found using self-reported measures (27.4%) compared to those identified using a structured interview or both [1]. In Serbia, 21% of pregnant women were screened as positive for depression during pregnancy and 11% during the postpartum period [2]. It is also indicated that there is a distinction between early onset postnatal depression occurring within two months after childbirth and late postpartum depression occurring up to one year postpartum [3].

First-time mothers, i.e., primiparous mothers in a singleton pregnancy, are a particularly vulnerable group to the onset of perinatal depression [4]. It was observed that they have higher rates of depressive symptoms than women who already have a child [5]. There is little data on the prevalence of perinatal depression in primiparous mothers without a previously detected psychiatric disorder [6].

Current knowledge in the field rejects the previously dominant etiological connection between hormonal changes during the perinatal period and perinatal depression [7]. Hormonal changes are now understood as a contributing factor in vulnerable women and as a result of the complex interaction of mood disorders, genetics, epigenetics, and environmental and social factors [7].

Available research associates perinatal depression with anxiety and alexithymia (a specific deficit in processing and recognizing one’s own and other people’s emotions and differentiating emotions from bodily sensations) [2]. Also, certain authors point to the additional need to observe perinatal depression as part of the bipolar spectrum, therefore screening for hypomanic/manic symptoms is indicated [8].

Undiagnosed and consequently untreated perinatal depression has a significant impact on the mother’s mental health, her bonding with the child, and subsequent social, cognitive, and emotional development in children, as well as society as a whole [2].

The course of perinatal depression can be different, depressive symptoms can disappear spontaneously at any time, but it is considered that almost 33% of women develop their first depressive episode during pregnancy, and over 40% of prenatally depressed women are depressed a few months after childbirth with significantly increased psychiatric morbidity in later life [9,10].

An additional problem is that we do not differentiate the depression that arises in the period before childbirth and then continues after birth and, on the other hand, the appearance of new depressive symptomatology that appears after childbirth, which would be a true representation of postpartum depression. For this reason, our research aims to establish a valid estimate of the frequency and course of perinatal depression by distinguishing between new episodes and residual depression in order to obtain a valid estimate of the dynamics of onset and remission of depressive symptomatology in the perinatal period. This would contribute to a more accurate determination of the incidence of postpartum depression as a new pathology in the postnatal period. Our secondary aim is to determine whether sociodemographic and psychological variables can be associated with postpartum depression.

## 2. Materials and Methods

### 2.1. Study Design and Data Collection

**Figure 1:**
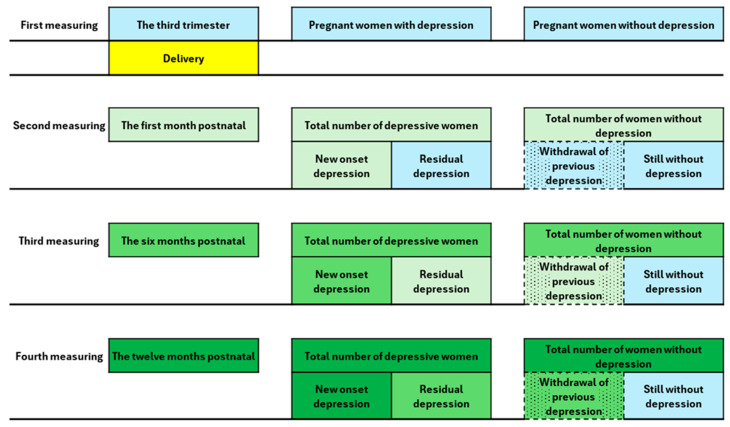
Flowchart of the research. The figure shows the flow chart of the research. The first column shows the sequence of the measurements. The second column shows the time when the measurement was taken (each measurement is marked with a different color). The third column shows the women with a depressive episode at the time of the measurement. This can be a depressive episode that was detected for the first time or a depressive episode that appeared in the previous period and persists during the new measurement (the last subjects are marked with the color of the previous measurement). The fourth column shows the women who were free of depression at the time of measurement. Women who were depression-free because their depression had regressed compared to the previous period are highlighted by a texture in addition to the color. In contrast, those who never developed depression during the study are highlighted in blue because their status has not changed since the first measurement.

The research was conducted at the Primary Health Center in Nis from January 2019 to June 2022 as a prospective, longitudinal, observational study during which the depressive symptomatology of pregnant/postnatal women was monitored at four time points: at the third trimester of pregnancy, and the first, sixth, and twelfth months postnatally (Figure 1). All procedures during this research were conducted following the Basics of Good Clinical Practice, the Declaration of Helsinki, as well as the Law on Health Care of the Republic of Serbia, with approval of the Medical Faculty Ethics Committee of Nis and the Primary Health Center Ethics Committee in Nis [11]. 

### 2.2. Study Setting, Sample, and Study Calculation

From January 2019 to July 2019, 350 pregnant women attending the Parenting School at the Department of Gynecology and Obstetrics at the Primary Health Center in Nis were offered participation in the study (from the 28th week of pregnancy and after checking the regularity of pregnancy by ultrasound examination). Only 251 accepted and returned the questionnaire (participation rate 71.71%; response rate 70.67%). After checking, 235 participants fully completed the questionnaire and were included in the study. In the end, we monitored 188 participants, because 15 were excluded due to a change in residence, four withdrew at the third time point, five at the fourth time point for personal reasons or at the insistence of a family member, and 23 respondents were excluded from the study because it was not their singleton pregnancy. The excluded pregnant women had similar partners and economic status and lived in the same areas as the women participating in the study. The sample size was calculated using the commercial statistical program G* power for two-sided null hypothesis tests. The following parameters were set to determine the size: Probability of error of the first type α = 0.05 and study power of 0.8. With these initial parameters and based on the publication by Dmitrovic et al. [2], in which the prevalence of postpartum depression in Serbia is reported to be 11%, a minimum representative sample size of 151 respondents was determined.

Inclusion criteria were first-time healthy pregnant/postnatal women in singleton pregnancy, without any previously detected depressive episode, history of psychiatric disorder and somatic disorder (primarily endocrinological, inflammatory, or autoimmune process), or application of drugs of any type that could explain the presented depressive symptomatology. All participants gave written consent, guaranteeing anonymity and data use exclusively for scientific and research purposes. 

### 2.3. Assessment Methods

All women completed the questionnaires at each measurement time point. All patients underwent a psychiatric/clinical examination at the beginning of the study to detect depression or other psychiatric diagnoses. In addition, all patients scoring 10 points or more on the Edinburgh Postnatal Depression Scale (EPDS) underwent a psychiatric examination, as EPDS must be used in conjunction with the clinical examination to confirm depression.

The survey constructed for research purposes included, among other things, age, place of residence, level of education, employment status, partner’s level of education, information about pregnancy and childbirth, family history, partnership, and emotional status, and subjective assessment of the relationship with the partner.

The risk of postnatal depression was measured by using a standardized and validated Serbian version of the Edinburgh Postnatal Depression Scale, widely used for detecting perinatal depression in our country [12,13,14,15]. EPDS is a self-reported instrument consisting of ten precisely structured questions for the detection of cognitive and behavioral symptoms of depression [12]. Each question is scored 0–3; the range is 0–30, with a clinical cut-off score set as 10 in Serbia [13]. In accordance with international guidelines and EPDS guidelines, the endpoint of the diagnosis of perinatal depression must be determined by a trained and certified psychiatrist with many years of experience [16,17,18]. Based on these two indicators—the cut-off score or more on the EPDS and the clinical data—we confirmed the presence of depression. For this reason, we used the term “depression” instead of “depressive symptoms”.

Toronto alexithymia scale (TAS-20) is a reliable alexithymia self-assessment instrument, standardized and used in PND research in Serbia [14,19,20]. The TAS-20 consists of 20 items in three subscales: Difficulty Describing Feelings subscale, Difficulty Identifying Feeling subscale, and Externally Oriented Thinking subscale, corresponding to the alexithymia construct. The total TAS-20 score indicates the general level of alexithymia. It represents the sum of responses to 20 items, i.e., three independent subscale results. The international cut-off values are as follows: no clinical picture of alexithymia (scores ≤ 51), borderline alexithymia (scores of 52–60), or alexithymia (scores > 60). In research, TAS-20 shows adequate convergent and concurrent validity [21].

Beck Anxiety Inventory (BAI)—It is an instrument for measuring anxiety and differentiating symptoms of anxiety from depression in the previous week [22]. The BAI consists of 21 items to which the respondent answers on a scale from 0 to 3, which are added together, where 0 means that the respondent does not have a specific symptom, and three means that the symptom is very pronounced. Based on the BAI, the level of anxiety can be expressed as mild (0–21), moderate (22–35), or severe anxiety (>35). The BAI is a frequently used instrument in daily clinical practice, takes 5 min to administer, and is intended for persons ≥18 years of age [22]. It has proven to be a reliable instrument for measuring anxiety in samples of psychiatric patients, healthy adults, and women in the perinatal period [23].

The Mood Disorder Questionnaire (MDQ) is a screening instrument for self-assessment of disorders from the bipolar spectrum, especially bipolar affective disorder type 1 and 2, that is, for symptoms of hypomania and/or mania [24]. It consists of a total of 15 questions. Most (the first 13) are answered with yes/no, and they assess mood, self-confidence, energy, sociability, interest in sex, and other behaviors, i.e., symptoms and the severity of impaired functioning caused by those symptoms. At the same time, the last two questions refer to life and family history [25]. A result in the direction of the bipolar spectrum of disorders implies seven or more positive answers to the first 13 questions, a positive answer to the 14th question, and “Moderately severe problem” or “Severe problem” to the 15th question [24]. The MDQ questionnaire is a useful screening instrument for bipolar spectrum disorders during pregnancy and the postpartum period [26]. 

### 2.4. Statistical Methods

All data were statistically processed with the Windows operating system’s IBM SPSS statistical software (version 21). The research results are presented in tabular and graphic form. 

Where appropriate, data are presented as absolute frequencies, percentages, means, and standard deviations. We used a non-parametric Chi-square test to examine the differences or associations between categorical variables. The level of *p* < 0.05 was chosen for the statistical significance assessment. 

## 3. Results

### 3.1. Group Structure

The group consisted of 188 participants, predominantly from urban areas (94.1%), employed (70.7%), in a partnership relationship (91.5%). Most participants continued their education after mandatory education (76.1%). Almost 4/5 of the participants were 25–35 years old (79.3%) (Table 1).

### 3.2. Depressive Episodes

#### Global Assessment of Depression in the Examined Period

To begin with, we separated a group of participants who, during the entire research period, from the last trimester up until the 12th month postnatally, never met the criteria for the appearance, maintenance, or withdrawal of pre-existing depression (96 participants or 51.1%) and a group of those who had depression (92 participants or 48.9%). 

### 3.3. Prevalence of Depressive Symptomatology at Different Time Points

Furthermore, we wanted to determine the extent to which depressive symptomatology was present during each time point (new-onset depression or residual depression from the previous period), as well as the prevalence of participants without depression at that moment (no current episode or withdrawal of depressive symptomatology from the previous period) (Figure 1). 

Based on the obtained results, there is a statistically significant increase in the number of participants with postnatal depression (X2 = 22.252 (3), *p* < 0.001). The number of participants with depression increased almost threefold (10.6% vs. 29.3%). The prevalence of participants with depression in the period from birth to one year does not change significantly (X2 = 0.883 (2), *p* = 0.643). Twelve months after childbirth, 25% of women met the criteria for a depressive episode (Table 2).

### 3.4. New-Onset Depression, Residual Depression, and Outcome

Given that we were also interested in new-onset depression, particularly postnatal depression, as well as the outcome of these episodes, for each period in which we performed the assessment, we determined the incidence of new-onset depression, prevalence of residual depression, as well as the prevalence of participants without depression, either because the previous episode withdrew or because it had never existed until that moment.

Based on the data obtained from our research, at one month postnatally, 25% of postnatal women developed a new-onset depression, i.e., postnatal depression. In the period from the first month to six months, 17% of women developed depression. In the last period, from six to twelve months, only 0.5% of women developed new-onset depression. In comparison to the previous period, the highest percentage of depression withdrawal was in the period from the first month to the sixth month after childbirth (20.7%). After that, we have a very modest percentage of withdrawal depression in the period from six months to twelve months—only 2.1% (Table 3).

### 3.5. Sociodemographic, Obstetric, and Psychological Factors and Perinatal Depression

Statistically significant sociodemographic, obstetric, and psychological factors are shown in Table 4. Being single with an unsatisfactory economic status, suicidality in the family, anxiety, alexithymia and absence of complications during childbirth were associated with new-onset, postnatal depression (*p* < 0.05). 

### 3.6. Bipolar Hypomanic/Manic Symptomatology at Each Time Point

In our sample, we did not find a significant increase in those with bipolar hypomanic/manic symptomatology compared to the last trimester of pregnancy (X2 = 3.336 (3), *p* = 0.343). During the follow-up period from one month to one year after delivery, there was a slight increase in patients with bipolar hypomanic/manic symptomatology, but without statistical significance (6.4% vs. 10.6%) (X2 = 2.190 (2), *p* =0.334).

## 4. Discussio

To our knowledge, this is the first prospective, longitudinal, and observational study on the prevalence, incidence, new-onset, and recurrence of depression using self-reporting and face-to-face interviews at different time points from the third trimester of pregnancy up until one year postnatally in primiparous healthy pregnant/postnatal mothers in a singleton pregnancy, without any previously detected depressive episode, history of psychiatric disorder and/or somatic disorder (primarily endocrinological, inflammatory, or autoimmune process), or application of drugs of any type that could explain the presented depressive symptomatology.

Based on our results, pregnancy, more precisely childbirth, is a period of high risk for depression. Almost half of the respondents had a certain level of depression in some period (from the last trimester up until one year postnatally), whether it was new-onset or the maintenance of a previously existing depression. It is also important to note that 51.1% were not depressed at any time during the follow-up.

The most significant increase in subjects with depressive symptomatology (new-onset and residual depression) was observed one month after giving birth when there was an almost three times higher percentage of subjects with depression compared to the last trimester (29.3% vs. 10.6%). The rate of global depressive episodes, either new or those remaining from the previous period, was reduced very slightly by the end of the study (12 months after childbirth). At the end of the research, 25% of the women still had depressive symptoms. The results of methodologically similar studies record a higher prevalence of depression during pregnancy than new-onset postnatal depression in studies in which prenatal depression was not observed as a risk factor for postnatal depression (12.4% vs. 9.6%) [9,27]. Studies that indicate the connection between depression in pregnancy and postnatal depression report a prevalence of 28.5% for prenatal and 27.6% for postnatal depression [2].

The highest percentage of new-onset depression is the first month after giving birth, and is maintained for up to six months, after which the appearance is sporadic. This means that the vulnerability to develop postnatal depression is higher in the first six months after childbirth, after which it decreases. This substantiates previous findings in the literature [28,29,30]. 

Given that the diagnosis of postnatal depression should be established at the end of the first month after childbirth, based on our data, the prevalence of new-onset postnatal depression was 25%, which is within the quoted prevalence of postnatal depression [9,28,29,30]. Lower percentages were mostly recorded among authors who used an EPDS score of 13 or higher, rather than a clinical interview to diagnose postnatal depression [9,26]. Higher prevalence values of perinatal depression were recorded among those with previous psychiatric disorders, especially depression, and women with low socioeconomic status having one or more children to look after [9,31].

When we take into consideration withdrawal from depression, the highest percentage was in the period between the first and sixth months after childbirth (20.2%). After that, the rate of depression was maintained over the period of the next six months (until the end of the investigation). That means that a new onset of depression and a withdrawal from depression are most intensive between the first and sixth months. This is in contrast with the data showing early onset postnatal depression occurring within two months after childbirth and late postpartum depression occurring up to one year postpartum [3]. One possible explanation is that the problematic postpartum period for a new onset of depression, postpartum depression, and resolution is within the first six months after delivery and that this period is the risk period for this separate type of depression. The rest of the depressive states that happen during the perinatal period probably belong to other types of depressive disorders. This hypothesis should be separately tested. The results were different in a similar study of maternal depression symptoms estimated during the first 21 months after giving birth. The prevalence at 9–12 months and 17–21 months is higher than the overall prevalence (27.8%: EPDS ≥ 12) [32]. We suppose that the rising prevalence of depression could result from different methodologies (longitudinal, prospective study vs. cross-sectional study; data collection by direct observation vs. web questionnaire). 

Based on our results, the dominant form of depressive episode in primiparous women is unipolar depression, but with the possibility of the development of bipolar disorder with manic/hypomanic symptoms later in life, which conforms to the available literature [8]. In our sample, in the third trimester, a relatively small percentage had a positive result on the MDQ scale for bipolar disorder. Given that the scale assesses the existence of hypomanic or manic symptomatology, in our group of respondents, only 6.4% probably had a hypomanic or manic episode, i.e., a potential bipolar disorder, during pregnancy. After giving birth, this relationship did not change, with the fact that by the end of the follow-up, more precisely, up to one year after giving birth, there was a slight but statistically insignificant increase in the number of women giving birth with signs of hypomania or mania (10.6%). Other studies also confirm an increase in the frequency of bipolar symptomatology during the perinatal period, but to a much greater extent—over 20% [8,33].

Our results suggest that the most important risk factor for new-onset postpartum depression was alexithymia in the third trimester and dissatisfaction with socioeconomic status. All women with these two variables developed a new episode of depression after delivery. In the current literature, alexithymia has been found to be an important predictor of postpartum depression. Additional variables have been found to mediate this relationship—self-criticism and self-compassion. Thus, psychological therapy that increases self-compassion and reduces self-criticism and alexithymia may be useful in preventing symptoms of postnatal depression [34]. In our study, a significant risk factor for new-onset depression was suicidality in the family and the absence of an emotional partner. Social support and dissatisfaction in marriage or with the partner are some of the most common social risk factors, along with stress and current or past abuse [35].

A significant strength of our research is that the observational period spans up to 12 months after childbirth. We have also separated women with a history of depression and/or postnatal depression from those with a first-ever episode during the perinatal period. We used EPDS as a screening instrument to confirm or to exclude a diagnosis with a cutoff of ten antenatally and postnatally, so we would not underestimate some cases with minor depression. Although several studies estimate the prevalence of non-psychotic postnatal depression, investigations on the distinction between new-onset and recurrent are poor.

Our study had its limitations. First, all the scales we applied are self-questionnaires. The assessment of depressiveness and depression was, however, different because an additional clinical evaluation by a psychiatrist confirmed it. The clinical evaluation was performed because the EDS is a self-questionnaire that gives us an indication of the presence of depressive symptomatology and the risk of having a depressive episode. Additional clinical evaluation confirms the diagnosis of unipolar depression. Also, considering that we included only women who had their first and singleton pregnancies in the research, our results cannot be generalized to the entire population of women in the perinatal period. Our study took place during the COVID-19 pandemic. Still, we did not separately analyze the differences between the period before the start of the pandemic and after, as there was only a tiny sample of respondents from the pre-covid period for statistical processing, as well as the available literature emphasizing that despite the increase in the frequency of mood disorders during the pandemic, the incidence and prevalence of PPD had not changed significantly [33]. Limitations of our study are the homogeneous socioeconomic status of our participants, as they were predominantly from urban areas, employed, in a partnership relationship, the voluntary nature of the participation in the study, and the fact that the excluded participants were significantly younger and more frequently unemployed, which seems to be associated with perinatal depression.

## 5. Conclusions

Depression in women during the perinatal period is very frequent. For a better understanding of the trajectory of depressive symptomatology and genuine postpartum depression, it is essential to classify them into those with new-onset and those who were left over from the previous observation period. Some depression starts at the prenatal period and continues after giving birth. One-quarter of women have genuine postpartum depression without previous depressive symptomatology. Our results imply that a new onset of depression is most intensive during the first six months, and after that, it is sporadic. Further studies are needed to explore whether all depressive symptomatology in the postnatal period is the same or perhaps postpartum depression, classified in this way, has specific characteristics, etiology, and consequently different treatment and prevention options.

## Figures and Tables

**Table 1:** Group structure.

		N	%
Place of residence	Urban area	177	94.1
	Suburban environment	11	5.9
Age	18–24	9	4.8
	25–35	149	79.3
	>35	30	16
Educational level	≤12 years of schooling	45	23.9
	>13 years of schooling	143	76.1
Employment	Employed	133	70.7
	Unemployed	55	29.3
Partnership status	Marriage/Cohabitation	172	91.5
	Without partner	16	8.5

**Table 2:** Prevalence of depression from third-trimester up to 1 year postpartum.

Depressive Episode	Last Trimester		One Month after Giving Birth		Six Months after Giving Birth		Twelve Months after Giving Birth	
	N	%	N	%	N	%	N	%
Present	20	10.6	55	29.3	50	26.6	47	25
Absent	168	89.4	133	70.7	138	73.4	141	75
Total	188	100.0	188	100	188	100	188	100

**Table 3:** The course of depressive episodes from the last trimester of pregnancy to 1 year postpartum.

		1 Month Postpartum	1–6 Months Postpartum	6–12 Months Postpartum
With depression	New-onset depression	25.0%	17.0%	0.5%
	Residual depression	4.3%	9.0%	24.5%
Without depression	Withdrew from depression	6.4%	20.7%	2.1%
	Without depression	64.4%	53.2%	72.9%

**Table 4:** Sociodemographic and psychological variables and PND.

		New-Onset Depression	Residual Depression	Withdrawal from Depression	Without Depression	X2	df	*p*
Partnership status	With partner	18.6%	8.1%	2.9%	70.3%	8833	3	0.032
	Without partner	50.0%	6.3%	0.0%	43.8%			
Economic status	Satisfactory	19.4%	7.8%	2.8%	70.0%	16,977	6	0.009
	Unsatisfactory	100.0%	0.0%	0.0%	0.0%			
	Already in debt	25.0%	25.0%	0.0%	50.0%			
Suicidality in family	Yes	71.4%	0.0%	0.0%	28.6%	11,036	3	0.012
	No	19.3%	8.3%	2.8%	69.6%			
Complications during childbirth	Yes	10.2%	5.1%	5.1%	79.7%	9482	3	0.024
	No	26.4%	9.3%	1.6%	62.8%			
Anxiety 1 month postnatal	High	17.4%	0.0%	0.0%	82.6%	15,634	6	0.016
	Moderate	37.5%	37.5%	0.0%	25.0%			
	Mild	21.0%	7.6%	3.2%	68.2%			
Alexithymia in the third trimester	Alexithymia	100.0%	0.0%	0.0%	0.0%	17,023	6	0.009
	Borderline alexithymia	56.3%	6.3%	0.0%	37.5%			
	No alexithymia	17.5%	8.2%	2.9%	71.3%			

## Data Availability

Data generated and analyzed during the study are available upon request to the author.
